# Supplementary material for: Low WT1 Expression Identifies a Subset of Acute Myeloid Leukemia with a Distinct Genotype
Source: Cancers (Basel). 2025 Apr 3;17(7):1213. doi: 10.3390/cancers17071213 (PMC11988028; doi:10.3390/cancers17071213)
Supplement: Supplementary file 1 [file cancers-17-01213-s001.zip › cancers-3416916-supplementary.pdf]

# Supplementary data

**Table S1: mutations in each patient**

| ID | gene | protein | c.DNA | type | codingConsequence | exon _rank | VF% |
|---|---|---|---|---|---|---|---|
| 1 | FLT3 | p.Gly583Arg | c.1747G>C | SNP | missense | 14 | 23,2 |
| 1 | ZRSR2 | p.Ser447_Arg448dup | c.1338_1343dupGAGCCG | INDEL | inframe_6 | 11 | 51,2 |
| 1 | ASXL1 | p.Gly646Trpfs*12 | c.1934dupG | INDEL | frameshift | 12 | 20,9 |
| 2 | TP53 | p.Arg273Cys | c.817C>T | SNP | missense | 8 | 76,2 |
| 3 | TP53 | p.Asn239Ser | c.716A>G | SNP | missense | 7 | 41 |
| 4 | TET2 | p.Gln491* | c.1471C>T | SNP | nonsense | 3 | 40,1 |
| 4 | TET2 | p.Gln1009* | c.3025C>T | SNP | nonsense | 3 | 41 |
| 4 | SRSF2 | p.Pro95His | c.284C>A | SNP | missense | 1 | 47,5 |
| 5 | SRSF2 | p.Pro95Leu | c.284C>T | SNP | missense | 1 | 39,1 |
| 5 | EZH2 | p.Ser695Leu | c.2084C>T | SNP | missense | 18 | 4,1 |
| 5 | CBL | p.Cys396Ser | c.1187G>C | SNP | missense | 8 | 35,3 |
| 5 | ASXL1 | p.Gly646Trpfs*12 | c.1934dupG | INDEL | frameshift | 12 | 33,9 |
| 5 | TET2 | p.His1756Asnfs*8 | c.5264_5265dupAA | INDEL | frameshift | 11 | 79,8 |
| 6 | SRSF2 | p.Pro95His | c.284C>A | SNP | missense | 1 | 37,6 |
| 6 | BRAF | p.Val600Glu | c.1799T>A | SNP | missense | 15 | 7,2 |
| 6 | ASXL1 | p.Ser846Ilefs*21 | c.2537delG | INDEL | frameshift | 12 | 4,1 |
| 6 | TET2 | p.Asp1750Glyfs*3 | c.5248dupG | INDEL | frameshift | 11 | 39,2 |
| 6 | TET2 | p.Cys1221Valfs*5 | c.3660delC | INDEL | frameshift | 6 | 43,2 |
| 7 | DNMT3A | p.Arg736Cys | c.2206C>T | SNP | missense | 19 | 36 |
| 8 | TET2 | p.? | c.4044+1G>C | SNP | splice_donor_+1 | 8 | 21,3 |
| 8 | EZH2 | p.Asp657His | c.1969G>C | SNP | missense | 17 | 67,4 |
| 8 | RUNX1 | p.Arg204Gln | c.611G>A | SNP | missense | 6 | 22 |
| 8 | IDH2 | p.Arg172Lys | c.515G>A | SNP | missense | 4 | 18,7 |
| 8 | TET2 | p.Gly1391Asp | c.4172G>A | SNP | missense | 9 | 39,7 |
| 8 | ASXL1 | p.Glu635Argfs*15 | c.1900_1922del | INDEL | frameshift | 12 | 40,7 |
| 8 | RUNX1 | p.Ser322*fs*1 | c.964_965insAGAT | INDEL | frameshift | 8 | 20,9 |
| 9 | EZH2 | p.Gln722* | c.2164C>T | SNP | nonsense | 19 | 3,4 |
| 9 | CEBPA | p.His195_Pro196dup | c.584_589dupACCCGC | INDEL | inframe_6 | 1 | 43,8 |
| 10 | PTPN11 | p.Asn308Asp | c.922A>G | SNP | missense | 8 | 20 |
| 10 | IDH2 | p.Arg140Gln | c.419G>A | SNP | missense | 4 | 22,7 |
| 10 | DNMT3A | p.Arg882His | c.2645G>A | SNP | missense | 23 | 20,1 |
| 10 | NPM1 | p.Trp288Cysfs*? | c.863_864insCAGC | INDEL | frameshift | 11 | 22,9 |
| 11 | ASXL1 | p.Gln546* | c.1636C>T | SNP | nonsense | 11 | 43,7 |
| 11 | SRSF2 | p.Pro95Arg | c.284C>G | SNP | missense | 1 | 45,6 |
| 12 | TP53 | p.Arg175Gly | c.523C>G | SNP | missense | 5 | 18,4 |
| 12 | TP53 | p.Gly226Alafs*21 | c.675delT | INDEL | frameshift | 7 | 18,3 |
| 13 | SRSF2 | p.Pro95His | c.284C>A | SNP | missense | 1 | 48,6 |
| 13 | NPM1 | p.Trp288Cysfs*? | c.860_863dupTCTG | INDEL | frameshift | 11 | 28,3 |
| 13 | ASXL1 | p.Asp756Thrfs*16 | c.2266delG | INDEL | frameshift | 12 | 48,8 |
| 13 | TET2 | p.Ser588*fs*1 | c.1763delC | INDEL | frameshift | 3 | 46,3 |
| 13 | TET2 | p.Leu1744Argfs*19 | c.5231delT | INDEL | frameshift | 11 | 48,3 |
| 14 | TP53 | p.Arg273His | c.818G>A | | missense | 8 | 83,6 |
| 15 | SRSF2 | p.Pro95Arg | c.284C>G | SNP | missense | 1 | 47,4 |

| | | | | | | | |
|---|---|---|---|---|---|---|---|
| 15 | IDH1 | p.Arg132Cys | c.394C>T | SNP | missense | 4 | 96,4 |
| 15 | DNMT3A | p.Arg882His | c.2645G>A | SNP | missense | 23 | 46,6 |
| 15 | FLT3 | p.Glu608_Asn609ins19 | c.1824_1825ins57 | INDEL | inframe_57 | 14 | 49,3 |
| 15 | RUNX1 | p.Ser322Phefs*? | c.964dupT | INDEL | frameshift | 8 | 47,3 |
| 16 | DNMT3A | p.Arg882His | c.2645G>A | SNP | missense | 23 | 43,8 |
| 16 | FLT3 | p.Lys623Ile | c.1868A>T | SNP | missense | 15 | 42,8 |
| 16 | NPM1 | p.Trp288Cysfs*? | c.860_863dupTCTG | INDEL | frameshift | 11 | 42,7 |
| 17 | RUNX1 | p.Leu56Ser | c.167T>C | SNP | missense | 4 | 48,8 |
| 17 | FLT3 | p.Asp835Tyr | c.2503G>T | SNP | missense | 20 | 6 |
| 17 | ASXL1 | p.Gly646Trpfs*12 | c.1934dupG | INDEL | frameshift | 12 | 7,1 |
| 18 | DNMT3A | p.? | c.1429+1G>A | | splice_donor_+1 | 11 | 48 |
| 18 | TET2 | p.Lys829* | c.2485A>T | | nonsense | 3 | 52,7 |
| 18 | PTPN11 | p.Asp61Val | c.182A>T | | missense | 3 | 39,8 |
| 18 | DNMT3A | p.Ile655Thr | c.1964T>C | | missense | 17 | 42,9 |
| 19 | U2AF1 | p.Gln157Pro | c.470A>C | SNP | missense | 6 | 16,1 |
| 19 | DNMT3A | p.Arg729Trp | c.2185C>T | SNP | missense | 19 | 24,3 |
| 19 | ASXL1 | p.Gly646Trpfs*12 | c.1934dupG | INDEL | frameshift | 12 | 12,9 |
| 19 | RUNX1 | p.Val132Aspfs*5 | c.395_396delTG | INDEL | frameshift | 5 | 7,1 |
| 19 | RUNX1 | p.Gly168Lysfs*10 | c.497_498insGTCAA | INDEL | frameshift | 5 | 5,9 |
| 20 | CEBPA | p.His195_Pro196dup | c.584_589dupACCCGC | INDEL | inframe_6 | 1 | 45 |
| 21 | IDH1 | p.Arg132His | c.395G>A | | missense | 4 | 13,8 |
| 21 | NRAS | p.Gly12Cys | c.34G>T | | missense | 2 | 12,1 |
| 21 | WT1 | p.Arg370Profs*15 | c.1109delGinsCC | | frameshift | 7 | 13,4 |
| 22 | TP53 | p.Arg273His | c.818G>A | SNP | missense | 8 | 53,2 |
| 23 | IDH2 | p.Arg172Lys | c.515G>A | SNP | missense | 4 | 38,5 |
| 23 | NRAS | p.Gly12Asp | c.35G>A | SNP | missense | 2 | 11,8 |
| 23 | NRAS | p.Gly12Ala | c.35G>C | SNP | missense | 2 | 4,9 |
| 23 | DNMT3A | p.Arg823Lys | c.2468G>A | SNP | missense | 21 | 39,9 |
| 24 | DNMT3A | p.Trp305* | c.914G>A | SNP | nonsense | 8 | 39,1 |
| 24 | EZH2 | p.Arg63* | c.187C>T | SNP | nonsense | 3 | 57,5 |
| 24 | RUNX1 | p.Arg201Gln | c.602G>A | SNP | missense | 6 | 36,4 |
| 24 | DNMT3A | p.Cys710Tyr | c.2129G>A | SNP | missense | 18 | 47,3 |
| 24 | TET2 | p.Gln891Thrfs*10 | c.2670dupA | INDEL | frameshift | 3 | 38,9 |
| 25 | NPM1 | p.Gln289Hisfs*? | c.867_876delGTGGAGGAA GinsCAGTCTTGGCGCCC | | frameshift | 11 | 11,6 |
| 25 | WT1 | p.Thr377Ilefs*9 | c.1129_1130insTCGA | | frameshift | 7 | 13,5 |
| 26 | KIT | p.Asn822Lys | c.2466T>G | SNP | missense | 17 | 26,1 |
| 27 | EZH2 | p.? | c.484+1G>A | SNP | splice_donor_+1 | 5 | 86 |
| 27 | RUNX1 | p.Arg320* | c.958C>T | SNP | nonsense | 8 | 71,9 |
| 27 | ZRSR2 | p.Glu109* | c.325G>T | SNP | nonsense | 5 | 82,6 |
| 27 | CEBPA | p.His195_Pro196dup | c.584_589dupACCCGC | INDEL | inframe_6 | 1 | 50,6 |
| 27 | ASXL1 | p.Leu775*fs*1 | c.2324delT | INDEL | frameshift | 12 | 42 |
| 27 | RUNX1 | p.Arg166Glnfs*10 | c.497delG | INDEL | frameshift | 5 | 5,1 |
| 28 | EZH2 | p.? | c.625+1G>A | SNP | splice_donor_+1 | 6 | 42,8 |
| 28 | KRAS | p.Gly60Val | c.179G>T | SNP | missense | 3 | 30,8 |
| 28 | NPM1 | p.Trp288Cysfs*? | c.860_863dupTCTG | INDEL | frameshift | 11 | 7,7 |
| 28 | ASXL1 | p.Pro647Trpfs*7 | c.1938_1948delCCCGGGT GGAG | INDEL | frameshift | 12 | 39,6 |
| 29 | DNMT3A | p.Trp306* | c.917G>A | SNP | nonsense | 8 | 39,4 |
| 29 | DNMT3A | p.Arg749Cys | c.2245C>T | SNP | missense | 19 | 43,5 |
| 29 | IDH1 | p.Arg132Cys | c.394C>T | SNP | missense | 4 | 41,1 |

| | | | | | | | |
|---|---|---|---|---|---|---|---|
| 29 | NRAS | p.Gly12Asp | c.35G>A | SNP | missense | 2 | 38,9 |
| 30 | SRSF2 | p.Pro95Arg | c.284C>G | | missense | 1 | 50,9 |
| 30 | NRAS | p.Gly12Ser | c.34G>A | | missense | 2 | 4,5 |
| 30 | NRAS | p.Gly12Asp | c.35G>A | | missense | 2 | 32,4 |
| 30 | TET2 | p.Pro410Leufs*17 | c.1229delC | | frameshift | 3 | 41,4 |
| 30 | TET2 | p.Ser1583Phefs*31 | c.4747dupT | | frameshift | 11 | 45,2 |
| 31 | SRSF2 | p.Pro95His | c.284C>A | | missense | 1 | 29,2 |
| 31 | IDH1 | p.Arg132His | c.395G>A | | missense | 4 | 30,3 |
| 31 | ASXL1 | p.Gly646Trpfs*12 | c.1934dupG | | frameshift | 12 | 31,1 |
| 32 | DNMT3A | p.? | c.1667+1G>A | | splice_donor_+1 | 14 | 41,4 |
| 32 | TET2 | p.Cys1193Trp | c.3579T>G | | missense | 5 | 40,8 |
| 32 | DNMT3A | p.Trp305Cys | c.915G>T | | missense | 8 | 42,9 |
| 32 | NPM1 | p.Trp288Cysfs*? | c.860_863dupTCTG | | frameshift | 11 | 37,8 |
| 32 | TET2 | p.Lys700Asnfs*4 | c.2100delA | | frameshift | 3 | 43,7 |
| 33 | DNMT3A | p.? | c.855+1G>A | SNP | splice_donor_+1 | 7 | 91,3 |
| 33 | PTPN11 | p.Glu69Val | c.206A>T | SNP | missense | 3 | 3,2 |
| 33 | FLT3 | p.Asp835Tyr | c.2503G>T | SNP | missense | 20 | 31,1 |
| 33 | FLT3 | p.Asp835Glu | c.2505T>G | SNP | missense | 20 | 6,6 |
| 33 | NPM1 | p.Trp288Cysfs*? | c.860_863dupTCTG | INDEL | frameshift | 11 | 40,7 |
| 33 | TET2 | p.Lys1090Argfs*16 | c.3269delA | INDEL | frameshift | 3 | 43,4 |
| 34 | IDH1 | p.Arg132His | c.395G>A | | missense | 4 | 51 |
| 34 | CBL | p.Arg420Gln | c.1259G>A | | missense | 9 | 52,6 |
| 34 | SRSF2 | p.Pro95_Arg102del | c.284_307del | | inframe_24 | 1 | 47,9 |
